# Influence of positive end-expiratory pressure on cyclic recruitment and derecruitment during one breathing cycle in porcine acute lung injury

**DOI:** 10.1186/cc13470

**Published:** 2014-03-17

**Authors:** IS Sulyok, A Johannes, S Bohme, KU Klein, J Baumgardner, O Kimberger, K Markstaller, R Ullrich

**Affiliations:** 1 Medical University of Vienna, Austria; 2 Oscillogy LLC, Folsom, PA, USA

## Introduction

Cyclic recruitment and derecruitment (R/D) of lung parenchyma during mechanical ventilation are responsible for atelectrauma. Theoretically, cyclic R/D can lead to varying degrees of pulmonary shunt fraction throughout the respiratory cycle. Measurements of dynamic pulmonary shunt fraction could help in assessing the degree of cylic R/D. However, common methods of measuring pulmonary shunt do not allow for dynamic serial measurements during one respiratory cycle. In this study, we measured serial dynamic pulmonary shunt fractions during one breathing cycle and investigated the effect of positive end-expiratory pressure (PEEP) on cyclic R/D in artificially ventilated pigs before and after saline washout induced acute lung injury.

## Methods

Pigs (*n *= 10) were anesthetized and ventilated at a tidal volume of 8 ml/kg and two levels of PEEP (0 and 15 cmH_2_O - conditions: PEEP0; PEEP15). Hemodynamic, respiratory and multiple inert gas analysis by micropore membrane inlet mass spectrometery (MMIMS- MIgEt; Oscillogy, USA) measurements were taken at PEEP0 and PEEP15 before and after saline washout. Retention of SF6, measured five times during one breathing cycle, was taken as a reflection of shunt fraction. MIGET sample acquisition was synchronized by electrical impedance tomography.

## Results

We observed dynamic changes in pulmonary shunt fraction, expressed as changes in SF6 retention, within all breathing cycles recorded, before and after induction of ALI. In healthy lungs at PEEP0 there was a decrease in SF retention at the end of inspiration and a return to baseline levels during expiration. In contrast, SF6 retention increased at PEEP0 in ALI during inspiration and decreased during expiration. In healthy pigs ventilated with PEEP15 there was an increase in SF6 retention at the end of inspiration and a return to baseline during expiration similar to the changes observed in pigs with ALI at PEEP0. In ALI at PEEP15, shunt decreased throughout inspiration and returned to baseline levels during expiration.

## Conclusion

Serial dynamic pulmonary shunt measurements during mechanical ventilation showed distinct variations over the respiratory cycle in both healthy and injured lungs. Increasing PEEP from 0 to 15 cmH_2_O altered the patterns of dynamic pulmonary shunt before and after ALI. Thus, serial assessment of dynamic pulmonary shunt fraction by SF6 retention with MMIMS-MIGET could prove useful for optimization of mechanical ventilation in healthy and injured lungs.

